# Ultraviolet vision in anemonefish improves colour discrimination

**DOI:** 10.1242/jeb.247425

**Published:** 2024-04-08

**Authors:** Laurie J. Mitchell, Amelia Phelan, Fabio Cortesi, N. Justin Marshall, Wen-sung Chung, Daniel C. Osorio, Karen L. Cheney

**Affiliations:** ^1^ School of the Environment, The University of Queensland, Brisbane, QLD 4072, Australia; ^2^ Queensland Brain Institute, The University of Queensland, Brisbane, QLD 4072, Australia; ^3^ Marine Eco-Evo-Devo Unit, Okinawa Institute of Science and Technology, Onna son, Okinawa 904-0495, Japan; ^4^ School of Life Sciences, University of Sussex, Brighton BN1 9QG, UK

**Keywords:** Colour vision, Psychophysics, Reef fish, Ultraviolet vision

## Abstract

In many animals, ultraviolet (UV) vision guides navigation, foraging, and communication, but few studies have addressed the contribution of UV signals to colour vision, or measured UV discrimination thresholds using behavioural experiments. Here, we tested UV colour vision in an anemonefish (*Amphiprion ocellaris*) using a five-channel (RGB-V-UV) LED display. We first determined that the maximal sensitivity of the *A. ocellaris* UV cone was ∼386 nm using microspectrophotometry. Three additional cone spectral sensitivities had maxima at ∼497, 515 and ∼535 nm. We then behaviourally measured colour discrimination thresholds by training anemonefish to distinguish a coloured target pixel from grey distractor pixels of varying intensity. Thresholds were calculated for nine sets of colours with and without UV signals. Using a tetrachromatic vision model, we found that anemonefish were better (i.e. discrimination thresholds were lower) at discriminating colours when target pixels had higher UV chromatic contrast. These colours caused a greater stimulation of the UV cone relative to other cone types. These findings imply that a UV component of colour signals and cues improves their detectability, which likely increases the prominence of anemonefish body patterns for communication and the silhouette of zooplankton prey.

## Teaser

**Summary:** A UV component of colour signals and cues improves detectability in anemonefish, which likely increases the prominence of their body patterns for communication and the silhouette of zooplankton prey.

## INTRODUCTION

Many animals have ultraviolet (UV)-sensitive (<400 nm) photoreceptors (reviewed by Tovée, 1995; Cronin and Bok, 2016), and UV vision contributes to behaviours including foraging (Church et al., 1998; Siitari et al., 2002), celestial navigation (Rossel, 1989; Homberg et al., 2011), mate selection (Bennett et al., 1996; Kodric-Brown and Johnson, 2002; Smith et al., 2002; Rick et al., 2006; Finkbeiner and Briscoe, 2021), individual recognition (Siebeck et al., 2010) and aggressive displays (Stapley and Whiting, 2006; Xu and Fincke, 2015). However, few studies have tested its contribution to colour discrimination, which is partly due to the technical challenge of producing UV stimuli (Powell et al., 2021), and it is unclear how UV sensitivity compares with that of visible range photoreceptors.

In vertebrates, cone photoreceptors in the retina mediate colour vision. Most teleost fishes, reptiles and birds have two morphological cone types: single cones and double cones. The latter is formed by the fusion of two cone cells (Engström, 1960; Marchiafava, 1985). Photoreceptor spectral sensitivity is primarily determined by its photopigment(s) comprising a G-coupled receptor opsin protein bound to a carotenoid-derived (vitamin A1 or A2) chromophore and can be modified by light filtering in the eye (Bowmaker, 1991; Lind et al., 2013).

Colour vision can be defined as the ability to discriminate colour by their spectral composition regardless of their relative intensity. This requires a comparison of signals from different spectral types of photoreceptors, typically by chromatic opponent neurons (Kelber et al., 2003). To fully encode spectral information, an eye with *n* spectral receptor types requires at least *n*–1 opponent mechanisms plus an achromatic (or luminance) mechanism (Kelber et al., 2003). The chromaticity (roughly hue and saturation) of a colour can be defined by its location within an *n*–1 dimensional colour space, such as 2-dimensional Maxwell's triangle or a tetrahedral colour space (Neumeyer, 1992).

UV contrast sensitivity has been reported in multiple animals, such as common goldfish (Neumeyer, 1992; Fratzer et al., 1994), zebrafish (Risner et al., 2006), budgerigars (*Melopsittacus undulatus*) (Goldsmith and Butler, 2005) and hummingbirds (*Selasphorus platycercus*) (Stoddard et al., 2020). Recently, the anemonefish, *Amphiprion ocellaris,* demonstrated an ability to detect UV targets (Powell et al., 2021), which did not strictly require colour vision and like the larval zebrafish (*Danio rerio*) (Yoshimatsu et al., 2020), a closely related anemonefish species (*Amphiprion akindynos*) has been found to have UV cones most concentrated in the frontal visual field (i.e. the centrotemporal retina) (Stieb et al., 2019). In larval zebrafish, the forward-looking retina forms a ‘strike zone’ (Yoshimatsu et al., 2020), which contains a high density of enlarged UVS cones, suited to detecting zooplankton prey but make little contribution to chromatic opponency (Yoshimatsu et al., 2020, 2021). Anemonefish UV/violet cones are also most abundant in a region of highest acuity or area centralis, but here, rather than being primarily for prey detection, UV sensitivity is thought to enhance the chromatic contrast of their UV-orange and UV-white colours for intraspecific communication (Stieb et al., 2019; Mitchell et al., 2023). However, it is unknown whether UV chromatic contrast provides any major benefit to colour discrimination in fishes or other species, despite the presence of ample UV in many environments.

Here, we tested the UV and non-UV colour vision capabilities of the false clown anemonefish, *A. ocellaris*. Anemonefishes (genus *Amphiprion*) are renowned for their symbiosis with sea anemones (Actiniaria) (Fautin and Allen, 1997) and their strict social hierarchy, which is determined by sex and body size (Fricke, 1979; Buston, 2003). In terms of their visual systems, anemonefishes were recently shown to have seven cone opsin genes, six of which are expressed in the adult *A. ocellaris* retina, where they produce four spectral types of cones (Mitchell et al., 2021a). However, the exact cone spectral sensitivities in *A. ocellaris* were unknown, as sensitivity estimations in Mitchell et al. (2021a) were made according to their assigned opsin(s). In this study, we first confirmed the sensitivity of all four cones using microphotospectrometry, and measured lens transmission.

We then conducted a behavioural experiment to examine how the UV single cones and the double cones contribute to colour discrimination. Specifically, we asked whether discriminability was better for colours with higher UV chromatic contrast (UV positive contrast) than those with lower UV chromatic contrast (UV negative contrast). To do this, we used nine different sets of test colours produced by an innovative five-channel (RGB-V-UV) LED display (Powell et al., 2021) customised to the *A. ocellaris* visual system, which allowed us to explore visual capabilities in UV regions of animal colour space. Behavioural discrimination thresholds were quantified using the receptor noise limited (RNL) model (Vorobyev and Osorio, 1998), which fits the psychophysical data of many species by assuming that colour discrimination only receives chromatic input and its lower limit is set by noise arising in the photoreceptors, rather than specific opponent mechanisms (see review by Olsson et al., 2018).

## MATERIALS AND METHODS

### Animals and ethics statement

Anemonefish (*Amphiprion ocellaris* Cuvier 1830; *N*=20) were acquired from a local aquarium store (supplier Gallery Aquatica, Wynnum, 4178 QLD, Australia). We used *N*=9 [female=3, mean (±s.d.) total length (TL)=4.5±0.5 cm; male=6, TL=3.5±0.5 cm) for taking measurements of cone spectral sensitivities, and *N*=11 (female=11, TL=4.9±0.3 cm) for behavioural experiments. Only females were behaviourally tested because of their boldness approaching the LED display which made them highly trainable; smaller males were timid and difficult to train. Previously, no clear differences in either the relative cone opsin expression levels or abundance of cone types were detected between the sexes of *A. ocellaris* (Mitchell et al., 2021a). Fish were housed individually in recirculating aquaria (60×30×30 cm) at The University of Queensland, and all experiments were approved by The University of Queensland's Animal Ethics Committee (QBI/304/16 and SBS/077/17). For anatomical measurements, anemonefish were euthanised by immersion in MS222 (500 mg l^−1^) for 10 min and subsequent decapitation.

### Lens transmission

For the measurement of lens transmission in *A. ocellaris*, the lenses (*n*=3 fish) were isolated from the hemisected eyecup and rinsed in PBS to remove any blood and vitreous. Spectral transmission (300–800 nm) was measured by mounting the lens on a drilled (1.0 mm diameter hole) metal plate between two fibres (50, 100 µm diameters) connected to an Ocean Optics USB4000 spectrometer and a pulsed PX2 xenon light source (Ocean Optics, USA). Light spectra were normalised to the peak transmission value at 700 nm, and the wavelength at which 50% of incoming light (*T*_50_) is transmitted was attained, as per Siebeck and Marshall (2001). No pigmented ocular media or cornea was observed.

### Photoreceptor spectral sensitivities

The spectral absorbance of *A. ocellaris* photoreceptors was measured using a computer controlled, single-beam, wavelength scanning microspectrometer (MSP). This procedure followed that outlined in detail elsewhere (see Cheney et al., 2009; Chung and Marshall, 2016). In summary, small pieces (∼1 mm^2^) of tissue were excised from the eyes of 2 h dark-adapted fish, then immersed in a drop of 6% sucrose (1×) PBS solution and viewed on a cover slide (sealed with a coverslip) under a dissection microscope fitted with an infra-red (IR) image converter. A dark scan was first taken to control for inherent dark noise of the machine and a baseline scan measured light transmission in a vacant space free of retinal tissue. Pre-bleach absorbance measurements were then taken by aligning the outer segment of a photoreceptor with the path of the measuring beam that scanned light transmittance over a wavelength range of 300 to 800 nm. Post-bleach scans were then taken after exposing the photoreceptor to bright, full spectrum ‘white’ light for 60 s, and then compared with pre-bleach scans to confirm the presence of a labile visual pigment. Confirmed visual pigment spectral absorbance data was then analysed using least squares regression that fitted absorbance data between 30% and 70% of the normalised maximum absorbance at wavelengths that fell on the long-wavelength limb. The wavelength at 50% absorbance was then used to estimate the maximum absorbance (λ_max_) value of the visual pigment by fitting bovine rhodopsin as a visual pigment template (Govardovskii et al., 2000; Partridge and De Grip, 1991). This absorbance curve fitting was performed in a custom (Microsoft Excel) spreadsheet, where the quality of fit of absorbance spectra between A1- and A2-based visual pigment templates was also visually compared. Individual scans were binned on their grouping of similar (≤10 nm difference) λ_max_ values, and then averaged and reanalysed across fish to create mean absorbance spectra (for individual measurements of photoreceptor spectral absorbance, see Figs S1–S3).

### LED display and stimuli calibration

**Fig. 1.**
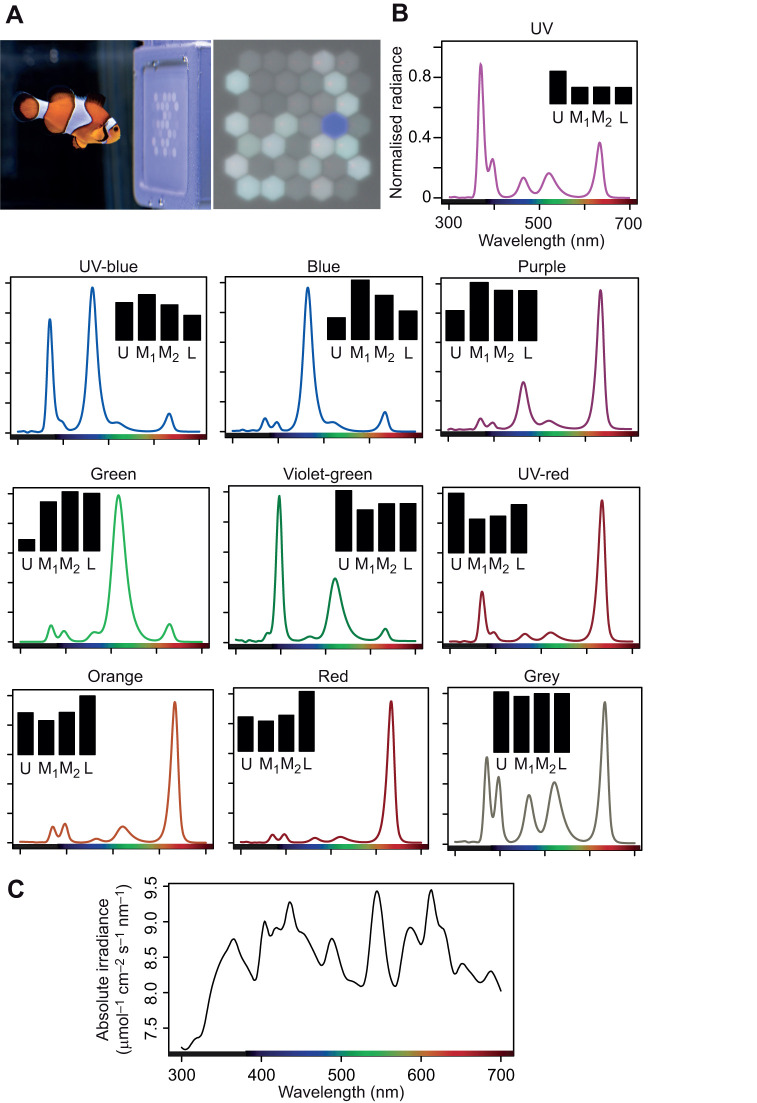
**Experimental setup for *Amphiprion*** ***ocellaris***. (A) Fish examining the RGB-V-UV LED display with an example stimulus. (B) The normalised spectral radiance of nominally saturated example targets and the average grey distractor. Bar plots depict the relative receptor stimulation (quantum catches) evoked by colours or grey for *A. ocellaris* against the PTFE screen of the LED display. Note that colour names have no implication for their appearance to the fish. (C) Side-welling measurement of the radiance flux (photons s^−1^ cm^−2^ nm^−1^) of the PTFE LED display screen/background under the aquarium lighting (fluorescent daylight tube and blacklight) in which colours were viewed. Note, *y*-axis units are in log scale owing to the steeply fluctuating fluorescent light that otherwise obscures the remainder of the spectrum. U, ultraviolet-sensitive; M_1_, medium-wavelength sensitive (MWS) 1; M_2_, MWS2; L, medium-wavelength sensitive (LWS); SWS, short-wavelength sensitive. Photo credit: Valerio Tettamanti.

To display the visual stimuli in our behavioural experiments we used a five-channel RGB-V-UV LED display (Fig. 1A; for full design details, see Powell et al., 2021). The five LED channels had peak emission values at 367, 395, 466, 526 and 629 nm. Note, that the violet channel (395 nm λ_max_) had an emission that emitted into the UV and violet, where it had higher overlap with the absorption curve of the UV cone but is referred to as ‘violet’ to distinguish it by name from the shorter wavelength ‘UV’ LED. The display itself was held within a waterproof, 3D-printed case, with a PTFE screen that acted as a light diffuser. A wide gamut of colours could be produced by modulating the relative outputs of each LED to colour mix the different channels (Fig. 1B).

Target and distractor colours were chosen to test anemonefish colour discrimination along nine different sets of chromatic contrast (Fig. 1B), including: UV colours (e.g. UV, UV-blue, violet-green, UV-red) and non-UV colours (e.g. blue, green, red, purple, orange). We first measured the spectral radiance (µmol l^−1^ cm^−2^ s^−1^ nm^−1^) of pixel colours using a spectrometer (Ocean Optics USB4000) with a 200 µm diameter UV-VIS fibre calibrated against a deuterium-halogen lamp (Mikropak DH2000-DUV, calibrated by Ocean Optics). An RPA-SMA holder (Thorlabs) maintained the fibre 1 mm directly above a pixel at a 90 deg angle.

The stimulus used for measuring discrimination thresholds was inspired by the Ishihara test of colour vision deficiency, as per Cheney et al. (2019). Anemonefish were trained to discriminate a target pixel which differed in chromaticity from distractors (Green et al., 2022). We ran the LED display via a Python script that pseudo-randomly assigned a target colour to one out of 38-pixel coordinates, while the 37 remaining pixels were assigned as grey distractors.

Analysis of target colour emission were performed using the ‘vismodel’ and ‘spec2rgb’ functions in the package PAVO2 (Maia et al., 2019; https://CRAN.R-project.org/package=pavo). This same package was used for calculating colour distances with the RNL model and plotted in a tetrahedral space.

#### Colour selection and stimuli design

To estimate anemonefish photoreceptor excitation for target and distractor colours, relative receptor quantum catches *q*, were first calculated for each stimulus, *S* (i.e. target and distractor radiance spectra in µmol l^−1^ cm^−2^ s^−1^ nm^−1^) viewed under well-lit conditions given by:
(1)

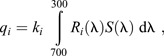
where *k* is a scaling coefficient for receptor adaption to the background ambient light, *S*_b_:
(2)

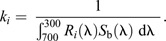


*R_i_*(λ) is the normalised spectral absorbance of a given receptor type ‘*i*’ [*i*=U (UV-sensitive), M_1_ (medium-wavelength sensitive, MWS1), M_2_ (MWS2), L (long-wavelength sensitive)] multiplied by lens transmittance and λ denotes wavelength (nm). *S*_b_(λ) is the spectral radiance of the PTFE display screen (between the pixels) with all LEDs turned off and measured from 5.0 cm in the experimental tank (Fig. 1C). This approach allowed for modelling spectral emission (from LEDs) rather than more commonly calculated for reflectance, as per Santiago et al. (2020). Integration was performed across the visible spectrum (i.e. 300–700 nm for *A. ocellaris*). Relative cone quantum catches (supporting data files) were used to plot colour loci in a tetrahedral colour space (as per Endler and Mielke, 2005; Stoddard and Prum, 2008).

Next, we calculated the chromatic contrast or colour distances (Δ*S*) of target colours relative to the average distractor spectra using the log receptor noise limited (RNL) model (Vorobyev and Osorio, 1998; Vorobyev et al., 2001). Further details of the RNL model and its equations can be found in the Supplementary Materials and Methods. A key assumption of the RNL model is that Δ*S* is determined by the differences in receptor stimulation elicited by two viewed stimuli, that is only constrained by receptor noise levels (*e_i_*) for each cone classes (*i*).

In the absence of direct noise measurements for *A. ocellaris* cones, we estimated cone noise levels (*e_i_*) by:
(3)

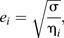
where σ, the numerator of the Weber fraction, is the coefficient of variance of noise within a photoreceptor cell (Vorobyev and Osorio, 1998; Olsson et al., 2015) and η is the ratio of the given cone type. We initially assumed that cone noise levels in *A. ocellaris* were like those reported in triggerfish and used a σ-value=0.05 (Champ et al., 2016; Cheney et al., 2013, 2019). Based on the regular mosaic of one single cone surrounded by four double cones in the *A. ocellaris* retina (Mitchell et al., 2021a), we used a relative cone abundance ratio of 1:2:1:1 (U:M_1_:M_2_:L) for a tetrachromatic visual system. The double cone ratio for the tetrachromatic scenario was based on *in situ* hybridisation experiments showing that *A. ocellaris* in our aquarium system express *RH2B* (M_1_) in one double cone member and either *RH2A* (M_2_) or *RH2A* with *LWS* (L) in the second double cone member (Mitchell et al., 2021a). Out of several models, we found that the best fit predicted receptor noise was σ=0.11 in the UV (single) cones, and σ=0.14 for the three types of double cone (as per Olsson et al., 2015; Lind et al., 2014) (all visual modelling is included in Fig. S4). We used more-conservative receptor σ-values ranging from 0.05 to 0.15, and compared threshold estimates between models of trichromat and tetrachromat vision in *A. ocellaris*, in case this could reveal any information on the contribution of double cones to colour vision. The closest model fit was determined based on which had the smallest mean difference summed across all colour lines from 1 Δ*S*. Although changing receptor σ-values caused shifts in the magnitudes of thresholds, it was found that the overall pattern in results remained consistent, where colours with higher UV LED input had lower discrimination thresholds.

Note that it is likely that colour thresholds are not set by noise in individual photoreceptors but depend upon pooling of receptor signals across a fixed area of the retina (Vorobyev and Osorio, 1998; Olsson et al., 2018). Consequently, thresholds should depend upon the density of a given receptor type in the retinal cone array. If relative receptor densities vary across the retina (e.g. Lind et al., 2014; Dalton et al., 2017), this might lead to variations in colour thresholds across the visual field.

We chose target colours that increased in Δ*S* away from grey distractors in nine different directions (Fig. 1B) within *A. ocellaris* colour space. Four of the nine colour set we collectively refer to by ‘UV colours’ (UV, UV-blue, UV-red, and violet-green) had increasing UV/violet LED emission, while the five remaining (blue, green, red, orange and purple) were ‘non-UV colours’ without increasing UV saturation. Four of the test colours also excited spectrally non-adjacent receptors more than intermediate receptors (violet-green, UV-red, orange and red), and so were non-spectral colours, i.e. the human equivalent of ‘purple’ (Stoddard et al., 2020), which could not be matched by a mixture of a monochromatic light with grey. 

Each colour set lay on a line radiating approximately from the central achromatic point in the anemonefish colour tetrahedron, so that they varied in saturation but not hue, and comprised of between 6 to 11 target colours (UV, *n*=10; UV-blue, *n*=6; blue, green, red, and violet-green, all *n*=9; purple, *n*=6; UV-red, *n*=11; orange, *n*=7). The target colour sets varied from a high-saturated target colour that was deemed highly contrasting against the grey distractors, to a low-saturated target colour that had low contrast (<1 Δ*S*) against the grey distractors.

Grey distractor spectra (*N*=13) were chosen to be <1 Δ*S* of the achromatic point of *A. ocellaris* and ranged between 0.3 Δ*S* to 0.8 Δ*S* of each other. Here, the achromatic point refers to equal stimulation of all photoreceptors. To control for the potential use of achromatic (intensity) cues when discriminating targets, we selected 6–10 distractor greys (from the 13) per stimulus based on all four-cone quantum catches to encompass the highest and lowest target intensities (see S2 Data in Mitchell et al., 2024).

#### Calculating hue angles

To gain additional information on the perceptual properties of colours including the sign direction of UV chromatic contrast and the presence of any complementary pairs, we calculated the elevation and azimuth angles of vectors plotted in anemonefish colour space which corresponded to the psychophysical thresholds of colour sets.

First, we converted Δ*S* to noise-corrected *xyz* Cartesian coordinates using the ‘jnd2xyz’ function in the R package PAVO2 (Maia et al., 2019), which performs calculations based on the algorithm from Maia et al. (2019) and Pike (2012). This returned *xyz* coordinates for colour threshold vectors representing the difference in receptor signal for the *x-*axis [L–(M_1_+M_2_)], *y-*axis [M_1_–(M_2_+L)] and *z*-axis [U–(M_1_+M_2_+L)].

We then calculated elevation angle Θ by:
(4)

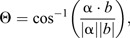
where α·*b* is the dot product of vectors α threshold *XYZ* position, and *b* threshold *xyz* position with the *z-*axis normal to the *xy* plane (i.e. zero) and |α| |*b*| are the magnitudes of each vector, e.g.:
(5)

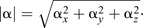


The product, in units of radians, was then converted to degrees and given appropriate signage to indicate relative position of Δ*S* above (positive) or below (negative) the *xy* plane (−90 deg≤Θ≤90 deg) of chromaticity formed by the double cones. Thus, giving an elevation angle where the vertex was at the grey point (origin) and position was relative to an (*xy*) equator to indicate UV receptor stimulation based on movement along the *x*-axis corresponding to the U cone direction.

The azimuth angle φ was calculated by:
(6)

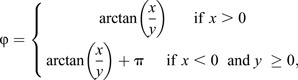
negative azimuth angles were corrected by adding 360 deg, so that all values ranged between 0 and 360 deg, where a bearing of 0/360 deg marked the *x*-axis describing L cone stimulation.

Because vectors are origin bound, the vectors representing colours that are situated on opposite sides of the achromatic point in anemonefish colour space (i.e. about 180 deg with each other on the *xy* plane) can be considered as complementary pairs. Complementary colours have minimal spectral overlap and excite distinct sets of photoreceptors, and when mixed in equal proportions, should be achromatic (i.e. they are equidistant from grey) (Cohen, 2001). This ‘horizontal’ or azimuth angle (0 deg≤φ≤360 deg) has an origin at the achromatic point and spans to the L cone at 0/360 deg with the U cone axis normal to the equatorial plane. Based on the approximately polar azimuth angles between some colour thresholds (φ±180 deg), we were able to identify two pairs of what are likely complementary colours: (1) green (φ=297 deg) and UV (φ=127 deg), and (2) purple (φ=128 deg) and violet-green (φ=298 deg).

### Training and experiment

Anemonefish were trained to peck a rewarded target pixel that differed in chromaticity from grey distractor pixels (Fig. 1A; Movie 1) (Cheney et al., 2019; Ishihara, 1917). During both training and the experiment, the LED display was presented in a section of the aquarium separated by a sliding, opaque door. This door was closed to keep fish from viewing the display while the stimulus was updated between trials, and only upon trial commencement was the door raised to allow fish to view and interact with the display. For both training and testing, a morning (09:00–11:00 h) and afternoon (14:00–16:00 h) session was run, in which fish completed between 10 to 12 trials per day.

Fish were initially enticed to peck the LED display by presenting a pseudo-randomly chosen high contrast pixel (blue, green, red or UV) with a small piece of prawn meat smeared on it. Over a week, we gradually reduced the size of the smeared food and transitioned towards a food reward (Formula One Ocean Nutrition pellets) delivered by forceps when fish pecked the single target pixel. Once anemonefish readily approached and pecked at the display without enticement, we introduced the grey distractor pixels alongside the target pixel (Fig. 1A). Fish were only rewarded when they correctly chose/pecked the target colour within 60 s. They were deemed to have reached the training criteria for the discrimination task after maintaining a correct choice probability of 0.75 over five consecutive sessions. Eleven anemonefish met this criteria (mean±s.d. number of training trials=8.0±4) and underwent experimental testing.

For testing, like training, fish were only rewarded for pecking the target pixel. Trials were terminated if fish made more than one incorrect choice or exceeded 60 s, upon which fish were returned to behind the divider (starting position) without reward. Note, because of the numerosity of pixels (*n*=38) per stimulus and the potential for distractions, each fish was permitted to make up to one incorrect choice per trial. For each trial, we recorded whether fish made a correct or incorrect choice, time (seconds) after fish entered through the door till target detection (i.e. latency), tested colour set, and target Δ*S*. Note that no significant differences in latency were detected between colour sets.

Each colour set was tested using five or six individual anemonefish that completed a minimum of eight trials per target colour per assigned set (mean±s.d.=10±1.0). Fish were divided into two groups assigned different colour sets, including: (1) fish IDs 19, 20, 33, 34 and 36, which were assessed in order of testing with green, UV, purple and UV-red, and (2) fish IDs 21, 22, 24, 31, 32 and 35, which were assessed in order of testing with blue, UV-blue, violet-green, red and orange.

Between each trial the target pixel contrast was pseudo-randomly assigned from a list of LED intensity values for each colour set. Throughout the experiment, we included control trials (*n*=10) to ensure that no other cues were created by the controller or code when choosing the target pixel, this determined the random chance of fish making a correct choice by displaying a target pixel of zero contrast (i.e. grey). In none of the control trials did fish correctly peck the control target.

To verify that differences in discrimination thresholds were not influenced by the order in which each of the colour sets were tested, we reassessed each of the nine sets at the end of the experiment using two anemonefish from each group. Although some fish had visibly steeper psychometric curves in the retest including for UV, UV-blue and blue, there was either no change or only minor differences in discrimination thresholds (range=0–0.4 Δ*S* shift, mean±s.e.m.=0.1±0.02 Δ*S* shift; see S1 Data in Mitchell et al., 2024).

### Software and statistical analyses

All statistical analyses and colour modelling were conducted using the statistical program R (v. 4.0.2) (https://www.r-project.org/). Psychometric curves fitted to individual fish data of correct choice probability for each target colour (Δ*S*) specified using the package ‘quickpsy’ (Linares and López-Moliner, 2016; https://CRAN.R-project.org/package=quickpsy). Discrimination thresholds were determined by the point at which fish had a 0.5 probability of making a correct choice, which was approximately at the inflection or steepest point of a sigmoid curve fitted to the behavioural data.

We first examined whether discrimination thresholds were different between colour sets using a linear mixed-effects model (LMM) run using function ‘lmer’ in the package ‘lme4’ (Bates et al., 2015; https://CRAN.R-project.org/package=lme). Individual threshold Δ*S* value was treated as the response variable, colour sets and the sign direction of UV contrast (positive/negative) were fixed factors, and fish ID was the random effect. A *post hoc*, pair-wise analysis controlled for multiple comparisons of threshold Δ*S* values across all possible combinations of colour sets using Bonferroni adjustment (p.adjust, R base package ‘stats’).

To test whether there were differences in how each colour set influenced the response of fish to the test and resulting shape of the psychometric curve, a generalised linear mixed effects model (GLMM) was run (Bates et al., 2015). Variables included anemonefish choice (0=incorrect, 1=correct) used as a binomial response variable, Δ*S*, colour set and the first-order interaction between the two variables treated as fixed factors and fish ID entered as a random effect. Model *P*-values were corrected for multiple comparisons via Bonferroni adjustment (‘p.adjust’, base R package ‘Stats’).

To validate model assumptions, residual diagnostics were performed for LMMs and GLMM using the package ‘DHARMa’ (https://CRAN.R-project.org/package=DHARMa), that checked the distribution of residuals and verified there were no dispersion issues in any of the models, and residuals were normally distributed in the LMMs.

Two-way ANOVA were performed which compared deviance in behavioural data from the RNL expected (1Δ*S*) threshold for tri- and tetra-chromatic models relative to individual fish variance in threshold. A *post hoc* analysis was performed using Fisher's least significant differences test, to make five specific comparisons between the predicted threshold and the mean behavioural threshold for each visual model.

## RESULTS

### Spectral sensitivities of *A. ocellaris*

**Fig. 2.**
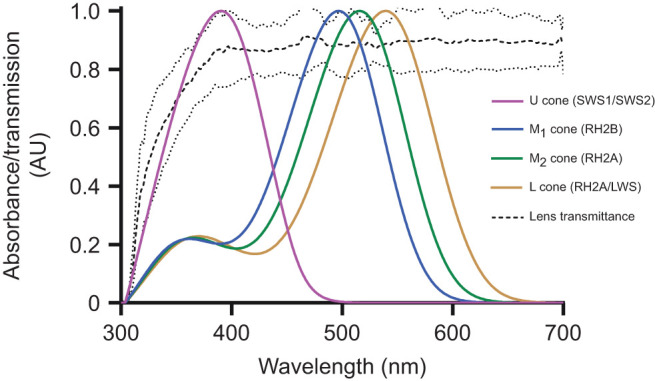
**Normalised average lens transmission and absorbance curves of the cones in anemonefish.** Dashed lines show lens transmittance and broken lines depict the upper and lower bounds of the s.d. (*N*=3 fish). Averaged cone absorbance spectra are shown in arbitrary units (*N*=9). In parentheses are the identities of (co-)expressed visual opsins that principally determine the spectral sensitivities of *A. ocellaris* cones (Mitchell et al., 2021a).

We first measured the lens transmittance and photopigment spectral sensitivities of *A. ocellaris*. The lens absorbed some UV wavelengths, with 50% transmission (*T*_50_) at 322, 340 and 341 nm in the three fish measured (mean *T*_50_=334 nm; Fig. 2).

Microspectrophotometry (MSP) of the cone pigments found one type of single cone (U) with a mean wavelength sensitivity of 386±5.0 nm λ_max_; Fig. 2). Three additional spectral cone types were double cones with λ_max_ values of about 497 nm (M_1_), 515 nm (M_2_) and 531/538 nm (L) (Fig. 2). These photoreceptors could be assigned specific visual pigments according to their previously identified opsin protein component (Fig. 2) (Mitchell et al., 2021a). One type of rod photoreceptor was present (mean λ_max_ value=502±4.0 nm; *n*=7 cells, *N*=4 fish) (Figs S1–S3).

All double cone absorbance spectra fitted a retinal (vitamin A1) derived chromophore visual pigment template. The single cone absorbance was considered due to the coexpression of UV- and violet-sensitive visual pigments, as has previously been shown to be the case in *A. ocellaris* (Mitchell et al., 2021a). *In vivo* photoreceptor spectral absorbance curves were given by the product of lens transmission and photoreceptor spectral absorbance measurements (Fig. 2). Two measurements hinted at a third MWS (M_3_) double cone type (508/509 nm λ_max_, *N*=1 fish), but the spectral overlap with the M_2_ cone meant it is unlikely that M_3_ makes a separate contribution to colour vision. No obvious differences in photoreceptor spectral absorbance were found between sexes; however, this could not be determined for the L cone because we only had two measurements.

### Colour discrimination thresholds

**Fig. 3.**
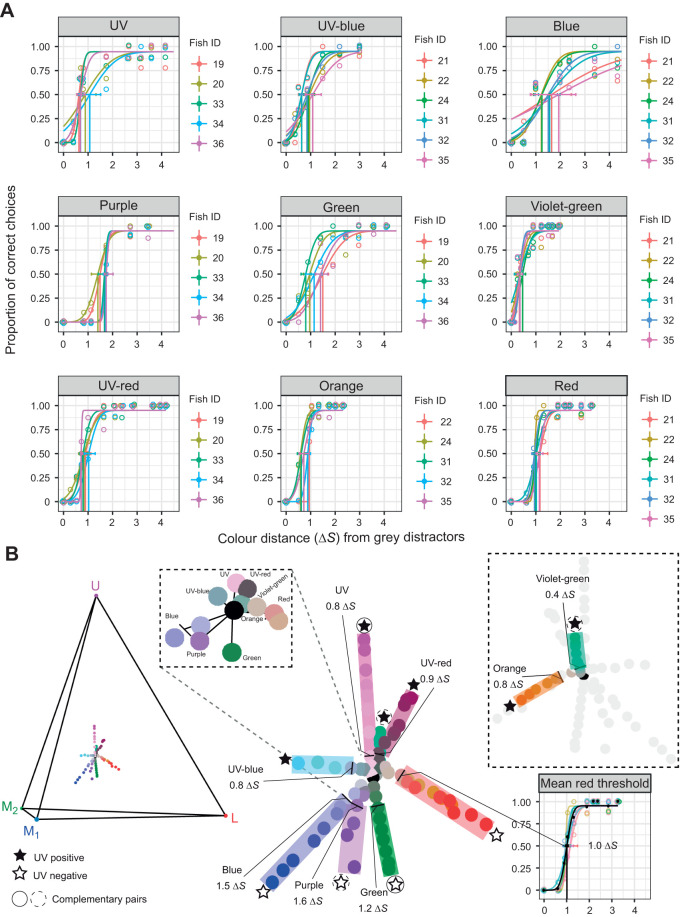
**Anemonefish colour discrimination thresholds and their hue angles in tetrahedral space.** (A) Colour discrimination thresholds shown as a function of the proportion of correct choices by anemonefish for targets with a range of chromatic contrasts (Δ*S*). Error bars denote 0.95 CIs. Discrimination thresholds are values calculated per fish (fish ID) and are indicated by vertical lines. Each plotted point represents the mean proportion of correct choices from one fish (*n*=8–17 trials). Note, the *x*-axis has been truncated to ≤4.5 Δ*S* for presentation purposes. (B) Relative positions of equal discriminability marked by intersecting lines in the receptor space of *A. ocellaris* for target sets of colours that varied in chromaticity (Δ*S*) in four principal directions representing the relative stimulation of the ultraviolet (U), medium-wavelength sensitive 1 (M_1_), medium-wavelength sensitive 2 (M_2_) and long-wavelength sensitive (L) photoreceptors. Given Δ*S* values are mean colour discrimination thresholds calculated per colour set from individual fish discrimination thresholds (an example shown by the black psychometric curve for the red set). Highlighted and non-highlighted regions indicate colours which were discriminable and non-discriminable from grey distractors, respectively. Star symbols and circles adjacent to colour sets indicate whether UV receptor input was needed for discriminating threshold colours from grey as determined by a positive elevation vector angle (Θ >0), and which colours potentially form complementary pairs according to polar azimuth vector angles (approximately ϕ±180 deg), respectively. Intersecting lines and adjacent numbers refer to the average discrimination threshold of each target set. The black dot represents the average grey distractor spectra which the receptor space is centred on (i.e. the origin). Insets include an enlarged view of the ∼1Δ*S* region surrounding grey with thresholds marked, and the reverse view of the receptor space to clearly show the orange and violet-green sets. Δ*S* was calculated by fitting behavioural data to the RNL model using a receptor noise coefficient (σ) of 0.11 for single cones and 0.14 for double cones. Note that colour schemes are unrelated across subfigures.

We conducted a total of 3921 test trials (*N*=11 fish, *n*=9 colour sets, *N*=84 colours, *n*=817 trials per target colour, mean=10 trials). Measured thresholds for the nine colour sets were: blue (mean±s.e.m=1.5±0.1 Δ*S*), purple (1.6±0.07 Δ*S*), green (1.2±0.1 Δ*S*), red (1.0±0.04 Δ*S*), orange (0.8±0.07 Δ*S*), UV-blue (0.8±0.07 Δ*S*), UV (0.8±0.09 Δ*S*), violet-green (0.4±0.02 Δ*S*) and UV-red (0.9±0.05 Δ*S*) (Fig. 3). Overall, discrimination thresholds were significantly lower for UV colours (positive UV contrast) compared with non-UV colours (negative UV contrast) [LMM, estimate UV^+^=−1.13±0.10 (mean±s.e.m.), *z*=−10.9, *P*<0.0001].

**Fig. 4.**
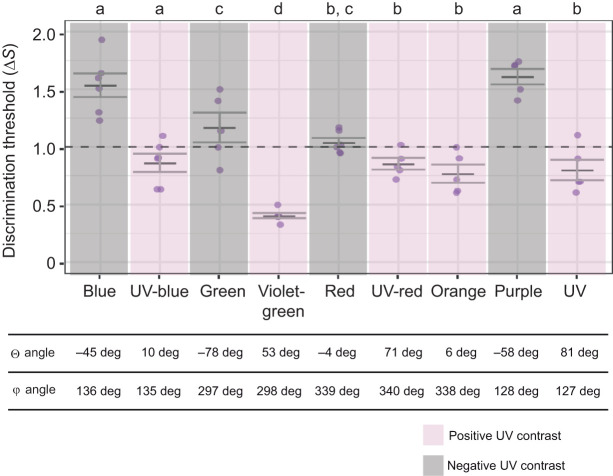
**Discrimination threshold fit comparisons among different colour sets.** Pairwise comparisons of *A. ocellaris* discrimination thresholds (Δ*S*) between colour sets with or without UV contrast. Letters denote statistical significance (*P*<0.05) between groups, as determined using a linear mixed effects model (LMM) with multiple paired comparisons. Individual points are discrimination thresholds calculated per fish (in Fig. 3A), and grey bars indicate mean values while error bars represent ±1 s.e.m. The dashed horizontal line indicates the RNL model assumed threshold (Δ*S*=1). Hue angles of colour discrimination thresholds in tetrahedral colour space, including elevation (Θ) and azimuth (ϕ) used to identify the sign direction of UV chromatic contrast (positive/negative) and complementarity between colours, respectively.

Comparing individual colour sets found that UV colours had significantly lower thresholds (UV=0.8 Δ*S*, Θ=81 deg; violet-green 0.4 Δ*S*, Θ=53 deg; UV-red 0.9 Δ*S*, Θ=71 deg; and UV-blue 0.8 Δ*S*, Θ=10 deg; Fig. 4) than three of the five non-UV colours (blue 1.5 Δ*S*, Θ=−45 deg; green 1.2 Δ*S*, Θ=−78 deg; and purple 1.6 Δ*S*, Θ=−58 deg) (LMM, all paired comparisons *P*<0.05; Fig. 4). Red (1.0 Δ*S*, Θ=−4 deg) and orange (0.8 Δ*S*, Θ=6 deg) had relatively low UV chromatic contrast in either sign direction and were located near the equatorial plane (−10 deg<Θ<10 deg) (Fig. 4). Both identified pairs of complementary colours had positive and negative UV chromatic contrasts, where the former had significantly lower discrimination thresholds for UV/green (LMM, estimate Δ*S*_UV–green_=−0.40, s.e.m.=0.11, *z*=−3.22, *P=*0.012; Fig. 4) and violet-green/purple (LMM, estimate Δ*S*_violet−green–purple=_−1.21, s.e.m.=0.11, *z*=−11.0, *P*<0.0001; Fig. 4).

Psychometric functions of the nine colour sets also differed significantly, with blue, purple, green, UV and UV-blue sets having more gradual functions (Fig. 3A) than orange, red, violet-green and UV-red sets (binomial GLMM, all *P*≤0.01). A more gradual incline was indicative of a higher error rate for relatively high Δ*S* targets, and a higher Δ*S* asymptote for discrimination performance. The differences in these functions were not attributable to the order in which colours were presented and followed no obvious pattern.

The modelled detectability of the test colour against the background (Δ*S*) strongly predicted choice latency for all colour sets except orange (LMM, all *P*<0.001; see S1 Data in Mitchell et al., 2024) but no significant differences were detected in the latency between colours.

We also compared the tetrachromatic model fit to that of four possible trichromatic models, where the input from one cone type was systematically dropped. Predicted discrimination thresholds in all four trichromatic models deviated significantly from the behavioural data relative to inter-fish variability (two-way ANOVA, range of estimated mean difference=0.101–0.40, *F*=18.1, all *P<*0.05). Whereas the tetrachromatic model (U-M_1_-M_2_-L) gave predicted discrimination thresholds that had minimal deviation from the data relative to inter-fish variability, and produced the closest fit (two-way ANOVA, estimated mean difference=−0.006, *F*=18.1, *P=*0.910). The next best fitting model was the U-M_1_-L model missing the M_2_ cone (two-way ANOVA, estimated mean difference=0.101, *F*=18.1, *P=*0.046).

## DISCUSSION

Using a newly developed LED display that allowed us to display both UV and non-UV colours to anemonefish, we highlight the potential importance of the UV receptor in anemonefish colour vision. We demonstrate that discrimination thresholds for UV colours (UV, UV-blue, violet-green and UV-red) were substantially lower than for non-UV colour sets (blue, green and purple). This systemic difference in discriminability was most convincingly shown by the complementary colour pairs UV/green and violet-green/purple, which are theoretically equidistant in Δ*S* from grey in colour space but had large disparities in psychophysical threshold distances. The asymmetry between colour discrimination thresholds cannot be directly attributed to noise in the early stages of the visual pathway such as photoreceptor noise or chromatic opponent neurons in the retina (i.e. the RNL model cannot explain this aspect of the data).

We confirmed that the anemonefish UV cone has a peak sensitivity (λ_max_) at 386 nm and contributes to putatively tetrachromatic colour vision with the three spectral types of double cones (λ_max_ 495 nm, 515 nm and ∼535 nm). This suggests that the cones containing all four combinations of the main pigment types (SWS1/SWS2, RH2B, RH2A, RH2A/LWS) (Mitchell et al., 2021a) contribute separately to colour vision, and that the UV cone has a comparatively high sensitivity. There is experimental evidence for tetrachromacy in a few other species, including goldfish (Neumeyer, 1992, 1986) and chicken (Osorio et al., 1999), but to our knowledge this is the first demonstration by testing the minimally saturated hues that can be distinguished from grey, which according to the closeness of RNL model predictions suggests that anemonefish have a 3D (tetrahedral) colour space. This advance was made possible by our five channel LED display customised to anemonefish vision (Fig. 1B). The display also allowed us to show that anemonefish can discriminate a wide variety of non-spectral colours from grey, which would be very difficult with monochromatic test lights (Stoddard et al., 2020).

While our modelling accounts for the relative abundance of cone types, it did not consider size differences in cone outer segments and the packing density of visual pigments which might impact photon capture. However, the outer segment of a single cone is shorter than those of double cones (Bowmaker, 1991), and in the anemonefish retina single cone (*sws1* and *sws2*) opsin gene expression is significantly lower than *rh2* and *lws* in double cones (Mitchell et al., 2021a), and therefore does not explain our results. One possibility is that activation of the UV receptor suppresses noise in the visual pathway (e.g. via temporal and/or spatial summation of cone signals (Vorobyev et al., 2001; Hawryshyn, 1991) or enhances the saliency of colours for anemonefish. The high sensitivity to violet-green, which was found in all six of the tested fish, is consistent with the heightened saliency of this colour.

It is unknown whether *A. ocellaris* also has a peak UV cone abundance in its centrotemporal retina; however, this seems quite likely given that they share multiple key features with their larger cousin *A. akindynos*, including similar cone spectral sensitivities and photopigment diversity (Stieb et al., 2019; Mitchell et al., 2021a), and common ecological aspects (life history, sea anemone habitat, social hierarchy, diet). In the zebrafish, UV cones in the centrotemporal ‘strike’ zone seem to make little input to colour vision (Yoshimatsu et al., 2020), while in anemonefish UV cones may have a disproportionately strong input; however, we cannot fully exclude the contribution of downstream processes (e.g. colour preferences). Nevertheless, this strong UV contribution to colour discrimination suggests that anemonefish colour patterns are very obvious to conspecifics and could benefit UV signalling used in social communication (Mitchell et al., 2023).

An innate (or learnt) UV preference in *A. ocellaris* could offer an alternative explanation for their acute discrimination, where a higher attention to UV might be influenced by the colour of their food or conspecifics. The white bars of *A. ocellaris* appear to have strong UV contrast against adjacent dark orange skin and sea anemone tentacles, as was shown to be the case in *A. akindynos* (Stieb et al., 2019). Indeed, juvenile anemonefish have a distinct UV colouration (studied in *A. akindynos*) shown to signal subordinance (Mitchell et al., 2023). Other suggested functions of anemonefish colour patterns include warning colouration (Merilaita and Kelley, 2018), camouflage (Merilaita and Kelley, 2018), species recognition (Salis et al., 2018) and mate recognition (Fricke, 1973). Future studies on the function of anemonefish colouration should include the UV and not restrict their spectral analysis to longer wavelengths in the human visible spectrum. Another potential basis for a UV preference in anemonefish could be to detect the UV contrast of their common prey (zooplankton) which can either scatter or absorb UV (Yoshimatsu et al., 2020; Novales Flamarique, 2013; Zimmermann et al., 2018). Larval anemonefish (*A. biaculeatus*) can solely rely on UV illumination (peaking at 365 nm) for detecting prey (Job and Bellwood, 2007), which might also be attributed to an achromatic UV channel as in larval zebrafish (Yoshimatsu et al., 2020). More generally, highly sensitive UV vision could help maintain the detectability of UV signals in habitats with reduced UV photon availability (e.g. deep water, dense foliage cover, heavy overcast), as suggested in goldfish (Hawryshyn and Beauchamp, 1985). Anemonefishes typically inhabit shallow coral reefs (∼115 m) where UV is abundant; however, a similar mechanism for facilitating acute UV discrimination might exist in other diurnal marine fishes and benefit UV vision in deeper habitats even beyond 100 m and at a maximum of 200 m (Losey et al., 1999).

An aversion towards blue and purple might explain the high discrimination thresholds (∼1.5 Δ*S*) and variable psychometric functions of anemonefish. This variation may indicate individual differences in learning (e.g. categorical perception), or differences in attentiveness and motivation. Guppy (*Poecilia reticulata*) also poorly discriminate purple because of possible neophobia (Sibeaux et al., 2019), and triggerfish have an aversion towards blue (Cheney et al., 2013), which was explained based on its common use as an aposematic colour, signalling unpalatability in some reef invertebrates (Winters et al., 2022). However, it should be noted that our experiment did not find evidence that fish were more (or less) responsive to any colour (i.e. the timing to peck targets did not vary across colour sets), nor were higher Δ*S* blue and purple targets avoided. Moreover, it is likely that any initial bias for a given colour would have been overridden by the training, which equally rewarded the colours.

An important point to consider is that our assessment of colour discrimination was limited to near the achromatic threshold, and whether the scale of these threshold differences persist elsewhere in colour space among highly saturated colours and shifts in hue remains a question for follow-up experiments using saturated (i.e. chromatic) distractors. Previous work using triggerfish found considerable variation in colour discriminability both near and away from the achromatic centre in their colour space (Green et al., 2022). The variable steepness of anemonefish psychometric curves might be an ‘early’ indication of heterogeneity deeper in anemonefish colour space.

One major aspect of anemonefish colour vision that remains unresolved is the exact nature of its cone opponency (i.e. the diversity of opponent cone interactions) for spectral encoding and whether this varies intraretinally. Work on larval zebrafish retinal circuitry has identified diverse cone opponency which can be roughly summarised as three main types of cone opponent circuits, of which one involves the UV channel (Baden, 2021). Moreover, the localisation of different cone pigments and/or opponent circuits in the teleost retina often varies dorsoventrally to match the visual field being sampled (i.e. downwelling light viewed ventrally, and upwelling light viewed dorsally) (Yoshimatsu et al., 2020; Baden, 2021; Dalton et al., 2014). Thus, distinct local forms of trichromacy can exist across the retina, and in theory, their combined effect could enable colour discriminability equivalent to tetrachromatic vision. Characterising the full range of opponent interactions across the anemonefish retina would clearly resolve whether they are functional trichromats or tetrachromats. This task would require the isolated stimulation of cones using intraretinal electrophysiological measurements with calibrated stimuli, multielectrode arrays and/or *in-vivo* calcium imaging. The considerable spectral overlap in anemonefish MWS double cones would also require the targeted silencing of individual double cone member input, for example, by using genome editing, which has produced ‘green’ opsin (RH2B) mutant *A. ocellaris* (Mitchell et al., 2021b).

## Supplementary Material

10.1242/jexbio.247425_sup1 Supplementary information
