# Supplementary material for: Ultraviolet vision in anemonefish improves colour discrimination
Source: J Exp Biol. 2024 Apr 8;227(7):jeb247425. doi: 10.1242/jeb.247425 (PMC11057877; doi:10.1242/jeb.247425)
Supplement: Supplementary information [file jexbio-227-247425-s1.pdf]

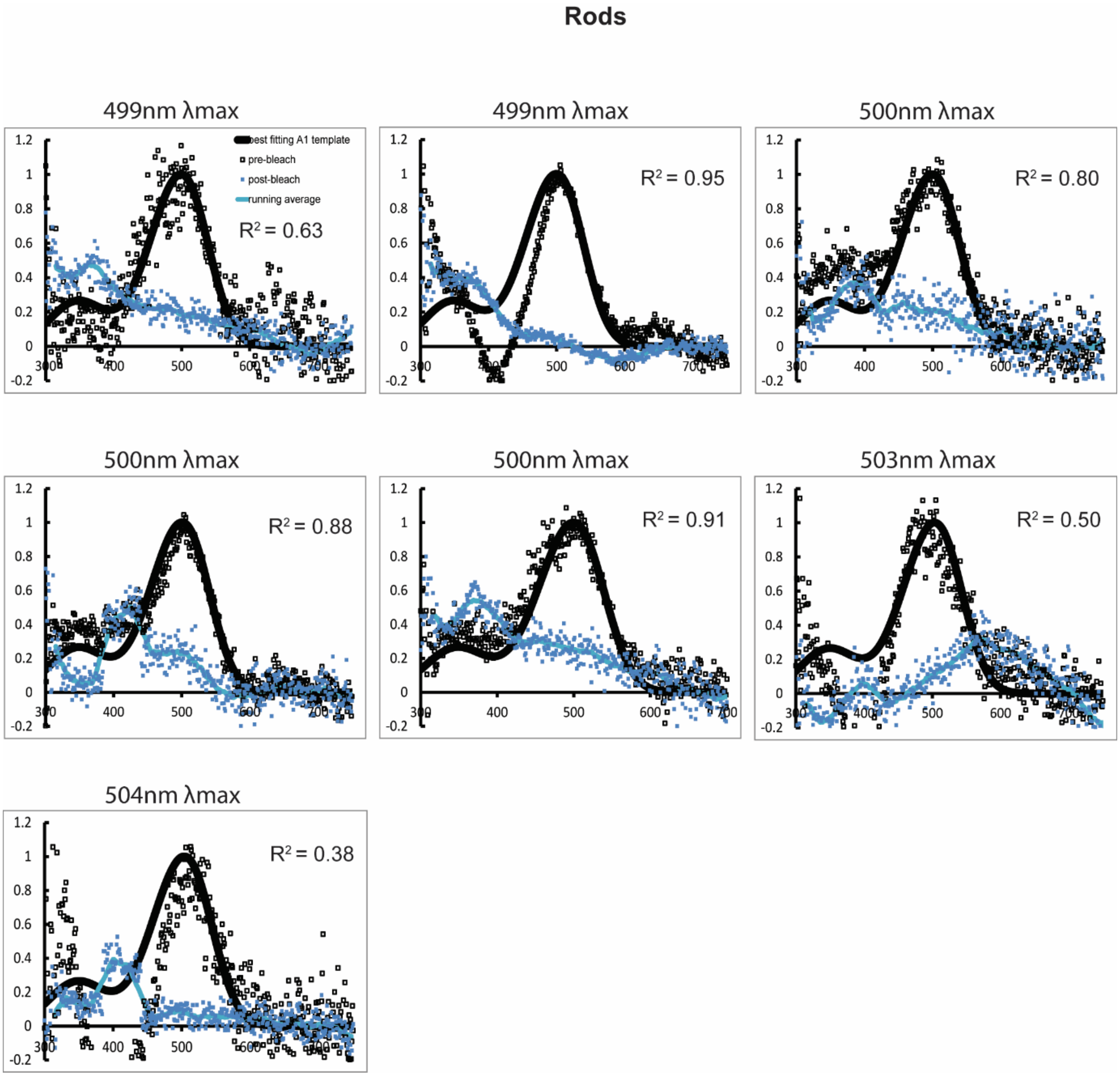


**Fig. S1.** Rod spectral absorbance measurements from microspectrophotometry. Normalised individual absorbance spectra from MSP for rod cells (n=7). Given are $\lambda_{max}$ values determined from individual scans and best fit $R^2$ values for an A1 visual pigment template. Each subplot contains pre-bleach absorbance spectra (black boxes) fitted with an A1 visual pigment template, and post-bleach absorbance spectra (blue boxes) overlayed with a running average curve (light blue).

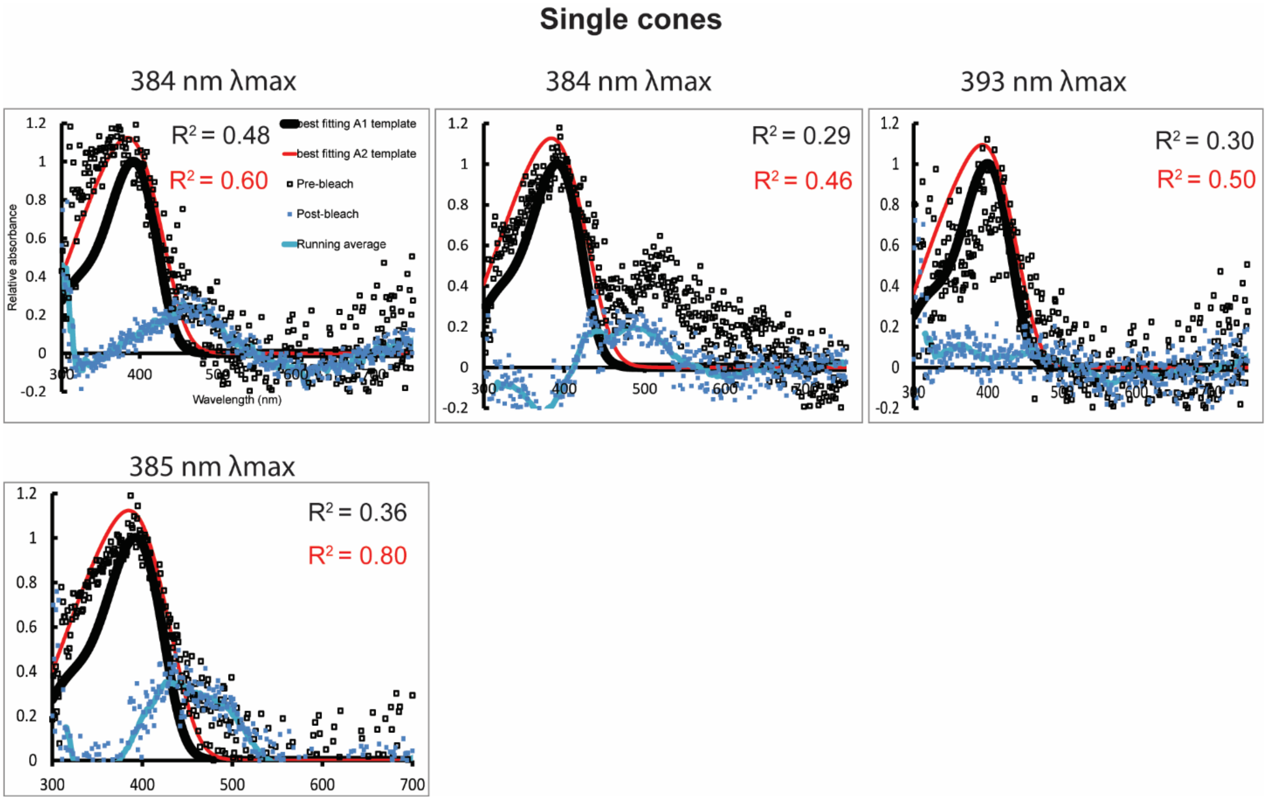


**Fig. S2.** Single cone spectral absorbance measurements from microspectrophotometry. Normalised individual absorbance spectra from MSP for single cones (n=4). Given are $\lambda_{max}$ values determined from individual scans and best fit $R^2$ values for both A1 (black) and A2 (red) visual pigment templates. Note that the A2 template is plotted as its broader curve can be a closer match to the spectral absorbance of cones which contain more than one (A1) visual pigment (in this case from the coexpression of SWS1/SWS2B). Each subplot contains pre-bleach absorbance spectra (black boxes) fitted with visual pigment templates, and post-bleach absorbance spectra (blue boxes) overlayed with a running average curve (light blue).

Journal of Experimental Biology • Supplementary information

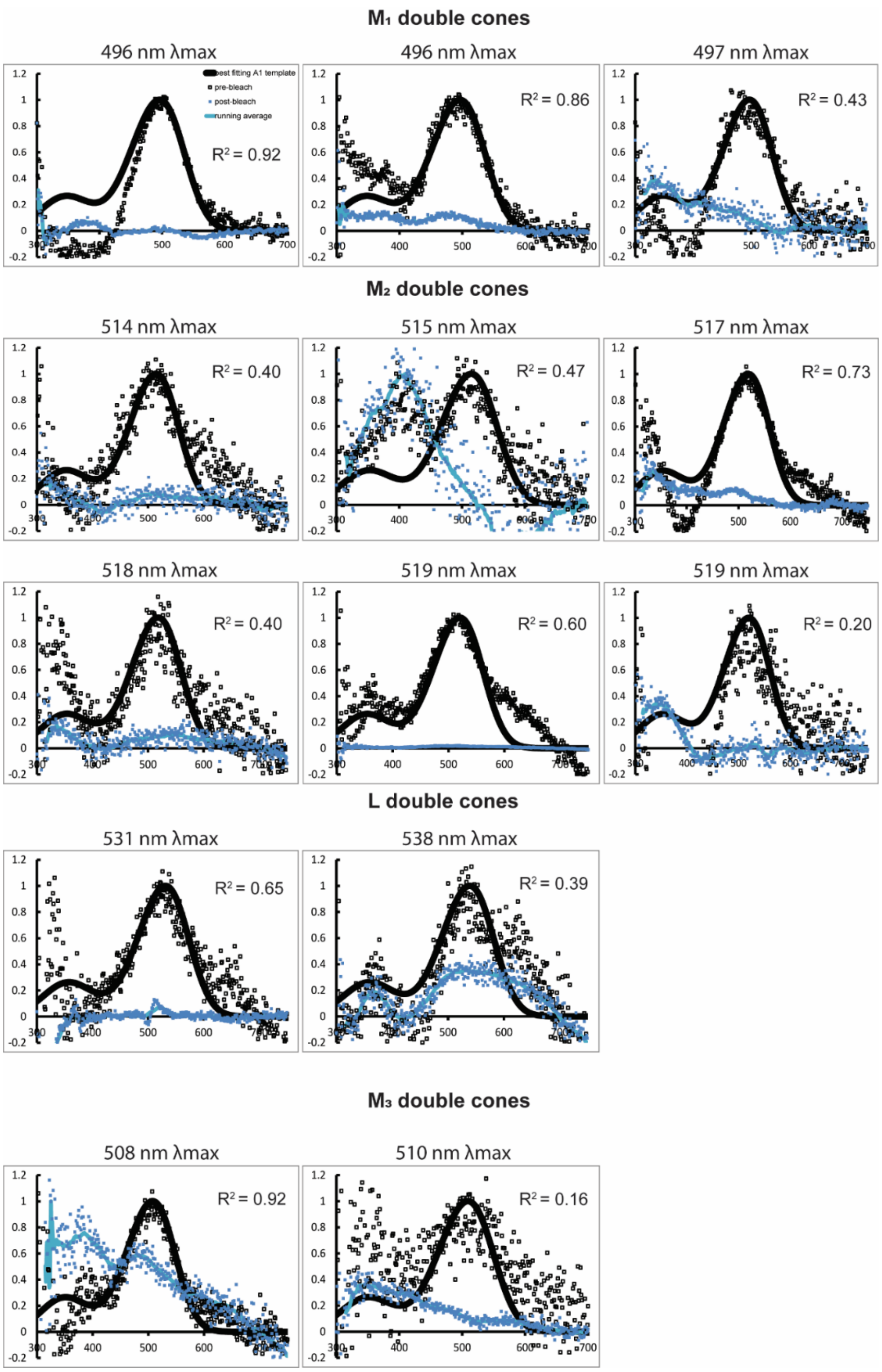


**Fig. S3.** Double cone spectral absorbance measurements from microspectrophotometry. Normalised individual absorbance spectra from MSP for double cones ($M_1$ n = 3, $M_2$ n = 6, L n = 2, $M_3$ n = 2). Given are $\lambda_{max}$ values determined from individual scans and best fit $R^2$ values for an A1 visual pigment template. Each subplot contains pre-bleach absorbance spectra (black boxes) fitted with an A1 (black) visual pigment template, and post-bleach absorbance spectra (blue boxes) overlayed with a running average curve (light blue).

Journal of Experimental Biology • Supplementary information

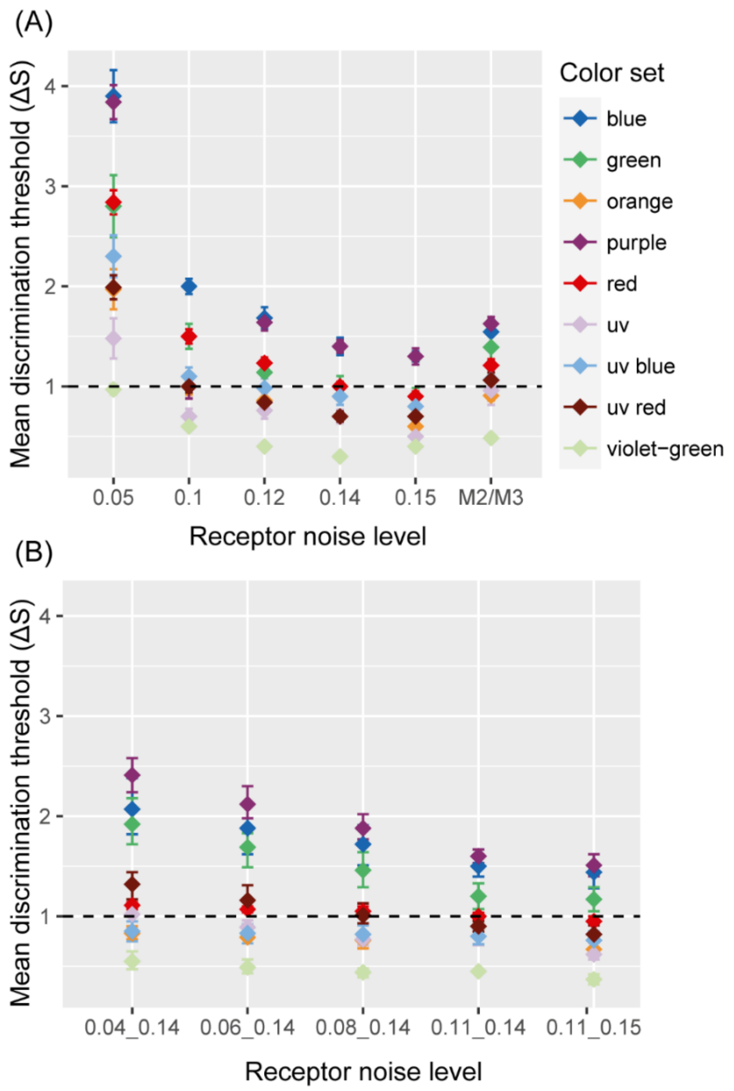


**Fig. S4.** RNL predicted color discrimination thresholds using different receptor noise levels. **(A)** Comparison of colour discrimination thresholds (Δ*S*) calculated using a range of receptor noise (σ) levels, with (or without) input by different MWS cones (using σ value = 0.14), and **(B)** additional distinct receptor noise levels for single cones and double cones. Initial and secondary receptor noise values ('X.XX_X.XX') refer to single cones and double cones, respectively. Discrimination thresholds were averaged across anemonefish for the Δ*S* which corresponded to a 50% proportion of correct choices. Error bars are the s.e.m. Broken, horizontal line demarks the RNL model assumed threshold (***S*** = 1). 'M2/M3' refers to modelling with the $M_3$ cone in place of the nearest spectrally neighbouring ($M_2$) cone.

Journal of Experimental Biology • Supplementary information

## Supplementary Materials and Methods

### Colour selection and stimuli design

The RNL model assumes: *1*) that 1 Δ*S* equates to a psychophysical threshold of one just noticeable difference between two stimuli of a given contrast, *2*) color vision is conveyed by chromatic mechanisms independent of achromatic visual processes, and *3*) that Δ*S* is determined by the differences in receptor stimulation ($\Delta q_i$) elicited by two viewed stimuli, that is only constrained by receptor noise levels ($e_i$) for each cone classes (*i* = 1, 2, 3, 4 for the U, $M_1$, $M_2$, L cone classes), or alternatively, for three cone classes in trichromat models (i = 1, 2, 3 for the U/$M_1$/$M_2$/L cone classes).

The contrast ($\Delta q_i$) for each receptor channel was calculated by,

$$\Delta q_i = \ln \frac{q_{i_{target}}}{q_{i_{average\ distractor}}} \qquad \text{(S1).}$$

Δ*S* in tetrachromatic visual space was calculated by:

$$\Delta S = \frac{(e_1e_2)^2(\Delta q_4-\Delta q_3)^2+(e_1e_3)^2(\Delta q_4-\Delta q_2)^2 +(e_1e_4)^2(\Delta q_3-\Delta q_2)^2+(e_2e_3)^2(\Delta q_4-\Delta q_1)^2 +(e_2e_4)^2(\Delta q_3-\Delta q_1)^2+(e_3e_4)^2(\Delta q_2-\Delta q_1)^2}{(e_1e_2e_3)^2+(e_1e_2e_4)^2+(e_1e_3e_4)^2+(e_2e_3e_4)^2} \qquad \text{(S2),}$$

and in trichromatic visual space was calculated by:

$$(\Delta S)^2 = \frac{e_1^2(\Delta_{q3}-\Delta_{q2})^2+e_2^2(\Delta_{q3}-\Delta_{q1})^2+e_3^2(\Delta_{q1}-\Delta_{q2})^2}{(e_1\,e_2)^2+(e_1\,e_3)^2+(e_2\,e_3)^2} \qquad \text{(S3).}$$

Journal of Experimental Biology • Supplementary information

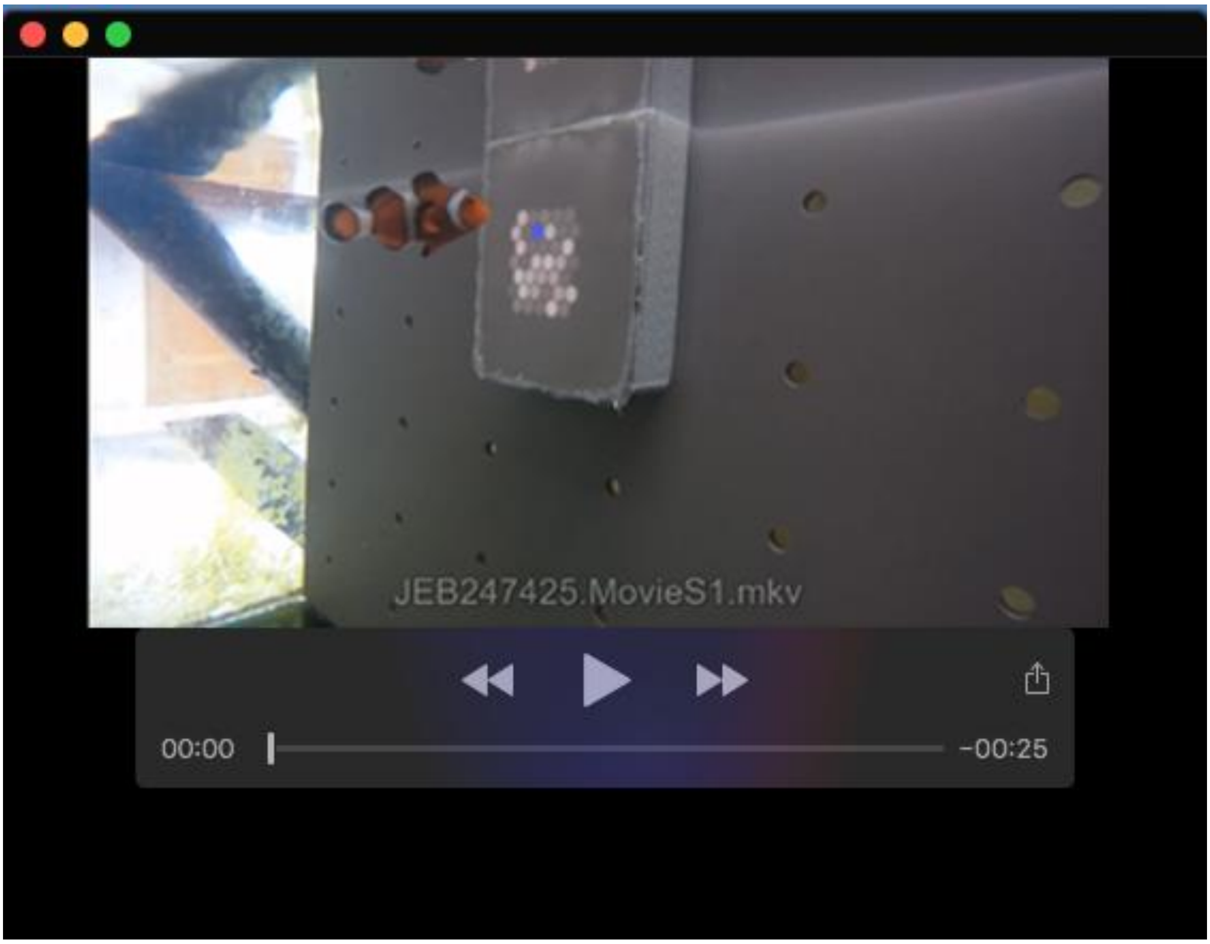


**Movie 1.** Anemonefish distinguishing a (blue) target from grey distractors on the LED display.

Journal of Experimental Biology • Supplementary information

## References

1. Olsson P, Lind O, Kelber A. 2015 Bird colour vision: behavioural thresholds reveal receptor noise. *Journal of Experimental Biology* **218**, 184–193. (doi:10.1242/JEB.111187)

2. Vorobyev M, Osorio D. 1998 Receptor noise as a determinant of colour thresholds. *Proceedings of the Royal Society B: Biological Sciences* **265**, 351–358. (doi:10.1098/rspb.1998.0302)

3. Champ CM, Vorobyev M, Marshall NJ. 2016 Colour thresholds in a coral reef fish. *R Soc Open Sci* **3**. (doi:10.1098/rsos.160399)

4. Cheney KL, Newport C, McClure EC, Marshall NJ. 2013 Colour vision and response bias in a coral reef fish. *Journal of Experimental Biology* **216**, 2967–2973. (doi:10.1242/jeb.087932)

5. Cheney KL, Green NF, Vibert AP, Vorobyev M, Marshall NJ, Osorio DC, Endler JA. 2019 An Ishihara-style test of animal colour vision. *Journal of Experimental Biology* **222**. (doi:10.1242/jeb.189787)

6. Mitchell LJ, Cheney KL, Lührmann M, Marshall J, Michie K, Cortesi F. 2021 Molecular Evolution of Ultraviolet Visual Opsins and Spectral Tuning of Photoreceptors in Anemonefishes (Amphiprioninae). *Genome Biol Evol* **13**. (doi:10.1093/gbe/evab184)

7. Olsson P, Lind O, Kelber A. 2018 Chromatic and achromatic vision: parameter choice and limitations for reliable model predictions. *Behavioral Ecology* **29**, 273–282.

8. Lind O, Chavez J, Kelber A. 2014 The contribution of single and double cones to spectral sensitivity in budgerigars during changing light conditions. *Journal of Comparative Physiology A* **200**, 197–207. (doi:10.1007/s00359-013-0878-7)

9. Dalton BE, De Busserolles F, Justin Marshall N, Carleton KL. 2017 Retinal specialization through spatially varying cell densities and opsin coexpression in cichlid fish. *Journal of Experimental Biology* **220**, 266–277. (doi:10.1242/jeb.149211)

Journal of Experimental Biology • Supplementary information
